# Unmeasured anions and mortality in critically ill patients in 2016

**DOI:** 10.1186/s40560-016-0171-2

**Published:** 2016-07-15

**Authors:** Yoshifumi Kotake

**Affiliations:** Department of Anesthesiology, Toho University Ohashi Medical Center, 2-17-6, Ohashi, Meguro, Tokyo, 153-8515 Japan

## Abstract

The presence of acid-base disturbances, especially metabolic acidosis may negatively affect the outcome of critically ill patients. Lactic acidosis is the most frequent etiology and has largest impact on the prognosis. Since lactate measurement might not have always been available at bedside, it had been regarded as one of the unmeasured anions. Therefore, anion gap and strong ion gap has been used to as a surrogate of lactate concentration. From this perspective, the relationship between either anion gap or strong ion gap and mortality has been explored. Then, lactate became routinely measurable at bedside and the direct comparison between directly measured lactate and these surrogate parameters can be possible. Currently available evidence suggests that directly measured lactate has larger prognostic ability for mortality than albumin-corrected anion gap and strong ion gap without lactate. In this commentary, the rationale and possible clinical implications of these findings are discussed.

## Background

Critically ill patients may suffer mixed acid-base disturbances, and therefore, the correct and timely evaluation and treatment of acid-base disorders is essential for the intensive care physicians. Additionally, the several compensating mechanisms exist in the body, and inadequate compensation might cause poor outcome. In that sense, the degree of primary acid-base disorder and adequacy of compensatory mechanisms may reasonably affect the mortality of the critically ill patients. There are two major approaches about the interpretation of metabolic component of acid-base status, i.e., physiological approach and physicochemical (Stewart) approach [1]. Each approach has its own advantages and limitations. Since Stewart approach provides more detailed and quantitative picture of acid-base status, it may be plausible to hypothesize that the acid-base status described by Stewart approach may provide better prognostic assessment [2].

## Main body

In this issue, Ho et al. published their retrospective analysis titled “A comparison of prognostic significance of strong ion gap (SIG) with other acid-base markers in the critically ill: a cohort study” [3]. Since SIG belongs to the parameters used in Stewart approach, many readers might have imagined that the results of head-to-head comparison between physiological approach and Stewart approach would be provided. However, the authors found no clear advantage of Stewart approach and concluded that lactate concentration played much greater role in the mortality than in acid-base parameters. Actually, the result is not unexpected since two previous studies reached similar conclusion [4, 5]. These results probably support the comment provided by Cuhaci in the accompanying editorial stating “It is probably fair to say that the likelihood of any prospective study to reveal additional important information on the association between any of these acid-base variables and the outcome seems to be remote.” [6]. However, I would like to comment on several issues about this topic.

First, lactate is a strong and measurable anion. Historically, both anion gap (AG) and SIG were developed to account for the contribution of unmeasured anions. AG is not strictly derived from the physiological approach but is typically used to determine the influence of anions other than bicarbonate in this approach. Thus, AG is comprised with weak anions such as charged (dissociated) albumin, phosphate, and lactate and other unmeasured anions. Alternatively, the contribution of dissociated albumin and phosphate are removed from the calculation of SIG; thus, the contribution of lactate and other unmeasured anions are represented in SIG. Then, lactate is almost fully dissociated in the body fluid and therefore should be regarded as strong anion. Additionally, most of the current blood gas analyzers in the ICU are capable of measuring lactate. Thus, the effect of lactate is typically removed from the calculation of SIG in the recent studies as Ho et al. did [3, 4]. Collectively, SIG without lactate theoretically represents the contribution of practically unmeasurable anions. Since the lactate acidosis represents one of the most typical acid-base disturbances in critically ill patients [7], it is quite reasonable that lactate has larger influence on the outcome than the other unmeasured anions.

Second, albumin is important in acid-base interpretation. The original definition of AG does not take into account the contribution of albumin. However, 75 % of AG is supposedly derived from negatively charged albumin in healthy state [8]. Hypoalbuminemia, typically seen in critically ill patients, has alkalinizing effect and makes the assessment of metabolic acidosis somewhat problematic. Therefore, the correction of AG by albumin (albumin corrected AG, ACAG) has been advocated for use [9]. By correcting AG with albumin, the difference between ACAG and SIG is theoretically the contribution of Ca, Mg, phosphate, and bicarbonate. Actually, the bias between ACAG and SIG is smaller than the bias between the original AG and SIG [10], and these two parameters probably significantly correlate as Cuhaci commented [6]. Collectively, it is also quite reasonable that ACAG and SIG have similar prognostic ability about mortality found in these studies.

Third, both hypo- and hyperchloremia are risk factor of unfavorable outcome. Chloride concentration has significant effect on acid-base status and such effect is more conveniently appreciated by Stewart approach. In the study by Ho et al. [3], there was little if any difference in the chloride concentration between survivors and non-survivors. On the contrary, Rocktaeschel et al. found significantly lower chloride concentration in non-survivors. Anyway, chloride concentration is not independently associated in increased risk of mortality in all the three studies regarding this topic [3–5]. However, as Ho et al. as well as others demonstrated [11], the U-shape relationship between chloride concentration and mortality probably exists. More studies accounting for such complex relationship between chloride and mortality are warranted. Besides, compensatory mechanism to metabolic acidosis caused by either lactate or unmeasured anions remains unknown. In the physiological approach, hyperventilation and renal reabsorption of bicarbonate comprise two major compensatory mechanisms against lactic acidosis. However, under the framework of Stewart approach, adaptation to increase strong ion difference is necessary to buffer the hydrogen ion derived from lactate. I personally hypothesize that successful compensation may be accompanied with some forms of chloride shift from plasma to other compartment, and this issue warrants further investigations.

## Conclusion

It is not surprising that acid-base disturbances at the ICU admission have limited impact on the mortality, partly because acid-base status is modifiable by interventions. In this sense, the effect of providing correct assessment and proper treatment on the outcome is more clinically relevant than the mere prediction of outcome at the admission of ICU. Every intensive care physician is recommended to make oneself familiar with the current approaches about acid-base physiology [1, 12–14].

## Abbreviations

ACAG, albumin corrected anion gap; AG, anion gap; SIG, strong ion gap
